# Nanostars in Highly Si-Doped GaN

**DOI:** 10.1021/acs.cgd.3c00317

**Published:** 2023-06-11

**Authors:** Marta Sawicka, Henryk Turski, Kamil Sobczak, Anna Feduniewicz-Żmuda, Natalia Fiuczek, Oliwia Gołyga, Marcin Siekacz, Grzegorz Muziol, Grzegorz Nowak, Julita Smalc-Koziorowska, Czesław Skierbiszewski

**Affiliations:** † Institute of High Pressure Physics, Polish Academy of Sciences, Sokołowska 29/37, 01-142 Warsaw, Poland; ‡ Faculty of Chemistry, Biological, and Chemical Research Center, University of Warsaw, Żwirki i Wigury 101, 02-089 Warsaw, Poland

## Abstract

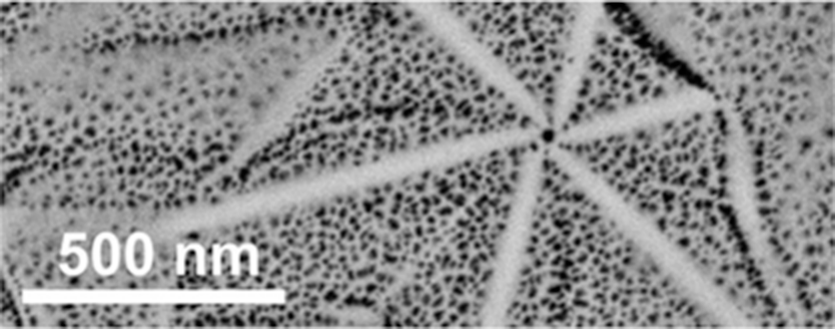

Understanding the relation between surface morphology during epitaxy of GaN:Si and its electrical properties is important from both the fundamental and application perspectives. This work evidences the formation of nanostars in highly doped GaN:Si layers with doping level ranging from 5 × 10^19^ to 1 × 10^20^ cm^–3^ grown by plasma-assisted molecular beam epitaxy (PAMBE). Nanostars are 50-nm-wide platelets arranged in six-fold symmetry around the [0001] axis and have different electrical properties from the surrounding layer. Nanostars are formed in highly doped GaN:Si layers due to the enhanced growth rate along the *a*-direction ⟨112̅0⟩. Then, the hexagonal-shaped growth spirals, typically observed in GaN grown on GaN/sapphire templates, develop distinct arms that extend in the *a*-direction ⟨112̅0⟩. The nanostar surface morphology is reflected in the inhomogeneity of electrical properties at the nanoscale as evidenced in this work. Complementary techniques such as electrochemical etching (ECE), atomic force microscopy (AFM), and scanning spreading resistance microscopy (SSRM) are used to link the morphology and conductivity variations across the surface. Additionally, transmission electron microscopy (TEM) studies with high spatial resolution composition mapping by energy-dispersive X-ray spectroscopy (EDX) confirmed about 10% lower incorporation of Si in the hillock arms than in the layer. However, the lower Si content in the nanostars cannot solely be responsible for the fact that they are not etched in ECE. The compensation mechanism in the nanostars observed in GaN:Si is discussed to be an additional contribution to the local decrease in conductivity at the nanoscale.

## Abstract 2

Highly doped GaN:Si layers, doped from 5 × 10^19^ to 1 × 10^20^ cm^−3^, were grown by plasma-assisted molecular beam epitaxy. Nanostars related to screw-type dislocations are observed in topography and electrical properties on the nanoscale. Electrochemical etching and Si mapping indicated lower incorporation of Si in the nanostar arms and decreased conductivity possibly due to the compensation by point defects.

## Introduction

Highly doped gallium nitride (GaN) layers are extremely important for many power electronic and optoelectronic devices: for effective current spreading, tunnel junctions, and low-resistance contact formation. The most common n-type GaN dopant is silicon (Si) as it readily substitutes gallium in GaN lattice, forming a shallow donor and allowing for ionization of almost all Si atoms at room temperature.^1,2^ Doping with Si is relatively straightforward because for the low and medium Si concentrations, the doping level in GaN:Si scales linearly with Si flux in plasma-assisted molecular beam epitaxy (PAMBE) and with SiH_4_ flow in metal–organic vapor phase epitaxy (MOVPE), respectively.^3,4^ Depending on the epitaxial technique used, the upper doping limits are slightly different.^5,6^ In case of PAMBE, efficient n-type doping with Si has been reported up to 2 × 10^20^ cm^–3^ and successful applications of such highly doped layers have been shown in laser diodes (LD) with tunnel junctions and LD stacks.^7,8^ For GaN:Si layers grown by MOVPE, a deterioration of material quality has been observed for Si doping of 6 × 10^19^ cm^–3^ as the increasing tensile strain in the layer leads to problems with surface morphology.^6,9−11^ Recently, germanium (Ge) has been intensively studied as an alternative to Si.^12^ Ge doping is advantageous in this regard as it allows to reach much higher concentrations than with Si, but it is not easy to avoid macro step formation of thick MOVPE grown GaN:Ge layers.^13^ In the case of MBE, smooth GaN:Ge layers are reported even for a doping level as high as 5 × 10^20^ cm^–3^.^14^ The concentration of Ge also scales linearly with the supplied atomic flux up to the point of surface degradation, and formation of Ge_*x*_N_*y*_ precipitates occurs.^15^ However, for MBE-grown Ge-doped layers that are grown under metal-rich conditions, it is important to note that the surfactant type, whether it is gallium or indium, dramatically impacts the doping interface abruptness.^14^ When GaN:Ge is grown with gallium as a surfactant, there is a tendency for Ge to stay in the gallium surface adlayer. Then, a memory effect for Ge doping is observed in the GaN layer grown on top even though the Ge flux is no longer supplied from the effusion cell.^14^ Abrupt doping profiles are achieved only for InGaN:Ge. In the case of Si doping, sharp doping profiles can be obtained both in GaN:Si and InGaN:Si that makes them of useful and applicable in device structures, therefore motivating the investigation of highly doped GaN:Si layers, their growth mechanisms, and finally, their electrical properties.

The homogeneity of the in-plane dopant incorporation at the nanoscale in planar layers is typically not discussed in the literature due to the lack of convenient tools giving access to dopant atom quantification in three-dimensional (3D) manner. The most typical methods used to access the information about the doping level are secondary ion mass spectroscopy (SIMS), Hall measurements, and capacitance–voltage (C–V). These techniques average out the in-plane concentration of doping atoms. The most common technique used to quantify the doping level in GaN structures is SIMS. High precision in depth-profiling and good reliability of the results make it a basic tool for calibrating the growth conditions for device purposes. Reaching a spatial resolution higher than 1 μm in SIMS has been shown possible in the example of oxygen decorating some screw and mixed-type dislocation cores.^16^ A more convenient technique to get an insight to local dopant atom distribution at a nanoscale seems to be energy dispersive X-ray spectroscopy (EDX). It has been used for instance to show the segregation of Si toward the edge of the of GaN:Si nanowires.^17^ Recently, we pointed out that electrochemical etching (ECE) can also provide information on local inhomogeneity in Si incorporation at the nanoscale since it is very sensitive to the doping level.^18^ Due to the fact that Si incorporation depends on the growth rate,^3^ the epitaxy on atomically flat surfaces was shown to provide uniform 3D Si distribution in GaN:Si layers, while in the case of the surfaces with step-bunching, the local differences in lateral growth rate of atomic steps imposed local inhomogeneity in Si incorporation. The ECE technique allowed us to trace the surface morphology evolution during epitaxy by the analysis of the pore-pattern on the cross section of the GaN:Si layer of interest.

GaN layers grown on foreign substrates, such as sapphire, contain a high threading dislocation density (TDDs) ranging from 10^8^ to 10^9^ cm^–2^.^19^ Screw and mixed type dislocations having a screw component of Burgers vector ***b*** = [0001] can promote the formation of growth spirals at the dislocation sites.^12,20^ As a result, when the GaN substrate misorientation angle is close to 0° from the (0001) plane, hillocks of six-fold symmetry are observed. The wurtzite symmetry of the GaN crystal lattice imposes the presence of double atomic steps that debunch and interlace at hillock arms along the *a*-direction ⟨112̅0⟩.^21,22^ Increased surface misorientation has been proven to be the most simple solution to suppress the formation of hillocks for Ga-polar (0001),^20,23^ N-polar (000-1),^24^ and m-plane^25^ GaN.

In this work, we show the evolution of the surface morphology of highly doped GaN:Si layers grown by PAMBE with increasing Si doping level from 1 × 10^19^ up to 1 × 10^20^ cm^–3^. High Si doping results in an increased lateral growth rate of single atomic steps along the *a*-direction ⟨112̅0⟩ that (1) promotes the hillock growth and (2) alters their hexagonal shape in a way that they resemble nanostars. Such nanostar-surface morphology is reported for the first time in highly doped GaN:Si layers grown by PAMBE under metal-rich conditions. Star-shaped patterns in crystalline solids were previously observed, e.g., in chemical synthesis of Au or PbS particles,^26−28^ arrangement of vanadium atoms in clusters on VTe_2_ surface,^29^ defects in CdZnTe layers,^30^ or in GaN after nanoindentation.^31^ However, these examples do not resemble the nanostars reported in this work because they are of a completely different origin.

The observed nanostars in GaN:Si consist of 50-nm-wide platelets (star arms) arranged in six-fold symmetry around the [0001] axis, with the arm length defined by local surface misorientation and the distance between adjacent hillocks. The surface of GaN:Si layers doped to the level of 5 × 10^19^ and 1 × 10^20^ cm^–3^ is covered by star-shaped hillocks as shown by atomic force microscopy (AFM) due to the high TDD in GaN/sapphire templates. Additionally complementary techniques such as ECE, scanning spreading resistance microscopy (SSRM), and EDX on transmission electron microscopy (TEM) specimens were used to examine the uniformity of Si incorporation in the highly doped GaN:Si layers with nanostars. We prove that the locally increased growth rate in the *a*-direction ⟨112̅0⟩ favors formation of star-shaped hillocks with arms that have slightly lower Si content. This decrease in Si content is quantified to be about 10% lower than the average Si content in the GaN:Si layer. Such low difference in the local Si doping level cannot solely explain the fact that the nanostars are not etched during ECE. Therefore, we expect a higher density of point defects or their complexes specifically in the nanostar arms. This hypothesis is additionally confirmed by SSRM that showed that the local conductivity of the nanostars is lower than the surrounding layer. Possible compensation mechanisms in the GaN:Si nanostars are discussed.

## Experimental Section

### Materials and Methods

The GaN:Si layers studied within this work were grown by PAMBE in custom designed VG Semicon V90 and Veeco Gen20A systems. Three 300 nm GaN:Si layers were grown with Si doping levels 1 × 10^19^, 5 × 10^19^, and 1 × 10^20^ cm^–3^. Layers doped to 5 × 10^19^ cm^–3^ and higher are denoted as GaN:Si^++^. Furthermore, a distributed Bragg reflector (DBR) structure consisted of 10×(46 nm GaN/66 nm GaN:Si *n* = 5.6 × 10^19^ cm^–3^) was grown. The structures were grown on Ga-polar (0001) GaN/sapphire templates prepared by MOVPE with misorientation angle ≈0.6° and dislocation density of ≈6 × 10^8^ cm^–2^. GaN:Si layers were grown under metal-rich conditions using Ga as a surfactant^32^ at 730 °C temperature. The growth rate was 0.36 μm/h. The Si doping level was calibrated by SIMS on separate GaN:Si structures in EAG Laboratories. The surface morphology was characterized by Nanoscope (Veeco Instruments) AFM. Prior to the AFM studies, the GaN:Si layer surface was cleaned in HCl to remove metal residuals after the epitaxy.

ECE was performed in 0.3 M oxalic acid. Schematics of the three-electrode setup used were presented in our previous work.^18^ Platinum plate is used as counter-electrode, and standard Ag/AgCl electrode is used as a reference. A positive potential is applied on GaN electrode, and as a consequence, a current flows through the GaN/electrolyte interface. After ECE, the pore morphology was assessed by scanning electron microscope (SEM) using Zeiss microscope at 4 kV. GaN:Si layers were etched from the top. In two samples, GaN:Si layer *n* = 1 × 10^20^ cm^–3^ and the DBR structure, the grooves were dry etched to allow electrolyte access and etch both from the side and from the top surface.

SSRM measurements of GaN:Si layer *n* = 1 × 10^20^ cm^–3^ were performed in contact mode using the NTGRA atomic force microscope with commercial diamond-covered tips. The sample was biased with a positive voltage of +3 V, and the current was collected between the tip and the electrical ground. Simultaneously, the corresponding topography image was collected. Current–voltage characteristics were measured locally at the selected points. Measurements were performed under dry nitrogen flow.

TEM studies were performed for the GaN:Si layer doped to the level of 1 × 10^20^ cm^–3^. Focused ion beam (FIB) was used to prepare cross-section specimen oriented in a way to perform the analysis along the *a*-direction ⟨112̅0⟩. Plan-view specimen was prepared by standard polishing and argon ion milling. TEM studies were conducted on a FEI Talos F200X transmission microscope at 200 kV. The measurements were performed in scanning TEM (STEM) mode using a high-angle annular dark-field (HAADF) detector. Composition measurements were carried out using EDX spectroscopy Super-X (Bruker BD4) with the approach proven previously to provide excellent precision in determination of local Si dopant concentration in nitride structures.^33^

## Results and Discussion

### Surface Morphology of GaN:Si Layers Grown by PAMBE under Metal-Rich Conditions

**Figure 1:**
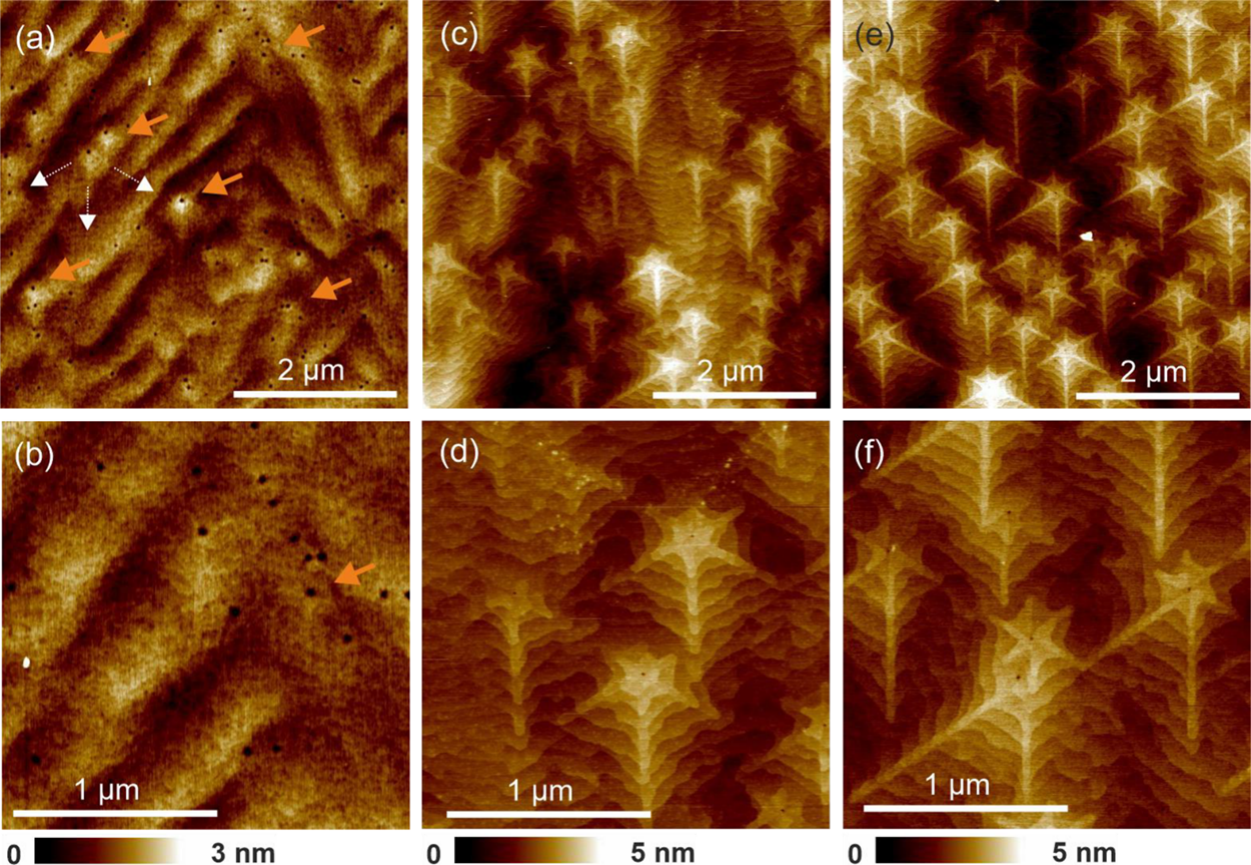
Surface morphology of GaN:Si layers with different doping levels: (a, b) 1 × 10^19^ cm^–3^, (c, d) 5 × 10^19^ cm^–3^, and (e, f) 1 × 10^20^ cm^–3^. The crystallographic orientation of all the images is the same, and the *a*-direction ⟨112̅0⟩ is marked with white arrows in (a).

The surface morphology of the GaN:Si layers doped to three different levels, 1 × 10^19^, 5 × 10^19^, and 1 × 10^20^ cm^–3^, is shown in Figure 1. Crystallographic orientation of all the images is the same, i.e., the *a*-direction ⟨112̅0⟩ is marked with white arrows in (a). For each layer, two AFM scan sizes are presented: 5 × 5 and 2 × 2 μm^2^. The height scale is presented below the images. Figure 1a,b shows the morphology of the GaN:Si layer with a doping level of 1 × 10^19^ cm^–3^. A wavy surface with step-bunching is observed. There is a high density of threading dislocations, visible as small holes, which is related to the fact that the growth was performed on GaN/sapphire templates. The density of holes in Figure 1b corresponds to the dislocation density of 4.3 × 10^8^ cm^–2^ that is a comparable value to the TDD in the GaN/sapphire template. The hillocks are not clearly visible, but the dislocations with faint contrast of elongated arms in the *a*-direction ⟨112̅0⟩ are marked with orange arrows.

Figure 1c,d presents surface morphology of the GaN:Si layer for Si doping of 5 × 10^19^ cm^–3^. The morphology of GaN:Si layer of the highest doping 1 × 10^20^ cm^–3^ is shown in Figure 1e,f. In both layers, the star-shaped hillocks with arms along the *a*-direction ⟨112̅0⟩ are clearly observed. The hillock density is 1.2 and 2.0 × 10^8^ cm^–2^ for the GaN:Si layers presented in (c) and (e), respectively. The arms are significantly higher than the background, ≈1–2 nm, and they are hexagonally arranged around the dislocation core. The nanostar arm width is ≈50 nm, while the arm length is related to the local substrate misorientation and is constrained by the neighboring hillock distance. Longer arms point out the direction of local surface misorientation and locally they exceed the length of 1 μm. Step edges at the apex are well resolved, proving that the hillocks originated at screw or mixed-type dislocations. Careful analysis of the hillock apex should allow in principle to estimate the length of the dislocation Burgers vector screw component.

### Pore Morphology of GaN:Si Layers after ECE

**Figure 2:**
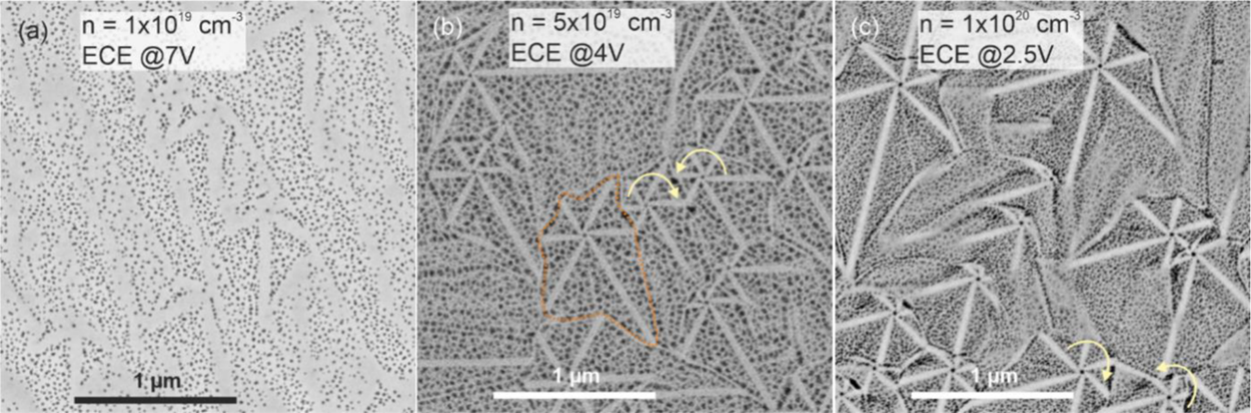
SEM images of the top surface of 300 nm thick GaN:Si layers after ECE. (a) GaN:Si *n* = 1 × 10^19^ cm^–3^ etched at 7 V, (b) GaN:Si 5 × 10^19^ cm^–3^ etched at 4 V, and (c) GaN:Si *n* = 1 × 10^20^ cm^–3^ etched at 2.5 V. The yellow arrows indicate two types of dislocation that are the sources of the growth hillocks, and the direction of the arrow is the direction of step propagation. The boundary of one hillock is marked with an orange dashed line.

SEM imaging of the GaN:Si layers after ECE was done to verify the local electrical properties at the nanoscale. Etching was carried out by exposing the top surface. Relatively low etching bias was selected to capture the differences in local doping and resolve the inhomogeneities in pore nucleation at the surface exposed to electrolyte. Figure 2 presents the SEM image of GaN:Si layers after ECE as viewed from the top. Pore morphology of GaN:Si with nominal average Si concentration of 1 × 10^19^ cm^–3^ after ECE at 7 V is shown in Figure 2a. It should be analyzed in reference to surface topography of this layer shown in Figure 1a,b. Surprisingly, despite the fact that the surface morphology of this layer is very typical for MBE-grown GaN on GaN/sapphire templates, the star-shaped features can be observed locally. This apparently “normal” surface morphology exhibits the fingerprints of tendency for increased growth rate along the *a*-direction as indicated by the orange arrows in Figure 1a. Non-uniformities in pore distribution related to the presence of step-bunching are also visible, that is in agreement with the surface morphology of this layer before ECE as presented in AFM image shown in Figure 1a,b. Note that other areas on this sample after ECE (not shown in this work) did not exhibit nanostar pattern, which is attributed to a locally higher surface misorientation that prevented hillock formation.

GaN:Si layer of higher doping of 5 × 10^19^ cm^–3^ after ECE at 4 V is shown in Figure 2b. Bright nanostars, related to star-shaped hillocks, are seen on the porous background. Pore size is relatively uniform. This SEM image corresponds to the surface morphology of this layer shown in Figure 1c,d. The boundaries of each hillock can be distinguished as marked with an orange dotted line to guide the eye. Interestingly, ECE revealed that there are two types of nanostars that resemble windmills rotating in two directions: clockwise and anticlockwise, marked with yellow arrows. The rotation direction is defined by the vertical component of the dislocation Burgers vector, which is along the +*c* [0001] or −*c* [0001̅] direction.

Figure 2c presents the SEM of GaN:Si layer of the highest doping 1 × 10^20^ cm^–3^ after ECE. The etching voltage in this case was 2.5 V, and it allowed to capture subtle peculiarities of the local conductivity at a nanoscale. In Figure 2c, the bright nanostars are again clearly seen. The pore size distribution in the area between the nanostars is as uniform as in Figure 2b. Boundaries between adjacent hillocks are preferentially etched. In some of the hillocks, there is an additional brighter line along the *m*-direction ⟨11̅00⟩, in the middle of the hillock side. This could be a fingerprint of the slightly different electrical properties being most likely the consequence of single atomic steps interlacing along the *m*-direction, that is, the case if hillocks that have 12 facets are grown.^34^

In summary, the pore formation in all highly Si-doped GaN layers under study is not uniform that indicates inhomogeneities in the Si incorporation into the layers or/and local incorporation of point defects, compensating the n-type conductivity. The most pronounce features after ECE are the nanostars that are not etched. Surprisingly, they are observed locally even in the sample that was doped to a relatively low level of 1 × 10^19^ cm^–3^, suggesting that some inhomogeneity in electrical properties is already expected despite no evident surface morphology features are seen in AFM studies.

**Figure 3:**
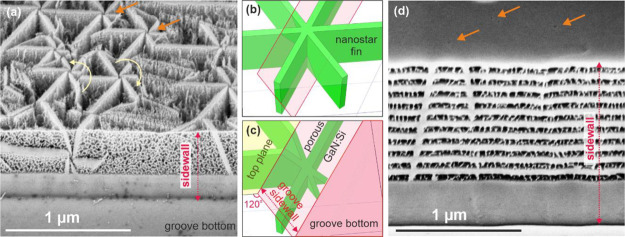
(a) SEM image of GaN:Si layer *n* = 1 × 10^20^ cm^–3^ etched at 3 V taken at 45°. Etching proceeded from the exposed sidewall of the groove and from the top. Nanostar cross-section is visible. The dislocation positions are marked with short orange arrows. Yellow arrows indicate the dislocations with the “+*c*” or “–*c*” screw component of the Burgers vector. (b) Schematics of a GaN:Si layer with nanostars. The inclined red plane denotes the groove sidewall and cuts the nanostar. (c) Nanostar cross-section by the groove plane that is inclined 120° in respect to the top surface. (d) SEM image of 10× GaN/GaN:Si DBR structure with *n* = 5.6 × 10^19^ cm^–3^ after etching at 5 V. Inhomogeneity in pore topography is caused by the nanostars that originated at the beginning of the epitaxy. The holes in the GaN surface layer are marked with arrows.

In order to get more insight on the 3D-distribution of Si inside the GaN:Si layers, grooves perpendicular to the surface through the epitaxial structure were formed by dry etching to allow lateral and vertical etching at the same time. The depth of the groove exceeded the thickness of the layer. Figure 3a presents the GaN:Si layer *n* = 1 × 10^20^ cm^–3^ after etching at 3 V as viewed at 45°. Sidewall of the groove is marked with a red dashed line. GaN:Si layer is not homogenously porosified. The nanostars are not etched, while the rest of the layer is highly porous. Each arm of the nanostar forms a sort of fin that is perfectly perpendicular to the sample surface and lies along the *a*-direction ⟨112̅0⟩, i.e., within the (11̅00) plane. The most important fact is that the nanostars are present in the full volume of the layer. Therefore, we can anticipate that the nanostars form at the beginning of the epitaxy of highly doped GaN:Si layer. Note that the groove sidewall is not perfectly perpendicular to the sample surface, but rather inclined at about 120°. The schematics of a nanostar embedded in GaN:Si layer and its cross section by an inclined plane are depicted in Figure 3b,c. The schematics explain that the star is seen at the sidewall due to inclined groove sidewall and not because it propagates along another growth axis. Lower porosity seems to be observed at the groove surface as compared to the top (0001) surface. This is in agreement with the previously reported lower etching rate of GaN:Si along non-polar directions as compared to the [0001] and [0001̅] polar directions.^35^ The yellow arrows indicate the nanostars associated with the dislocations having a “+*c*” or “–*c*” screw component of the Burgers vector.

Figure 3d presents the SEM image of the DBR structure consisting of 10× GaN/GaN:Si *n* = 5.6 × 10^19^ cm^–3^, after ECE at 5 V, viewed at 45°. Prior to etching, a groove across the whole structure was formed by lithography and dry etching. The top layer is undoped GaN, and therefore, it remains not etched during ECE. Small holes associated with the dislocations are visible and marked with orange arrows. Looking at the sidewall of the DBR structure after ECE, we see that the pore pattern in the GaN:Si layers is not uniform. Two ≈50-nm-thick unetched lines are visible: one across the whole structure and the other one crossing five top DBR pairs. Because the groove walls are not ideally perpendicular to the sample surface, these lines are inclined as already discussed above. The unetched lines are associated with the nanostars formed during the epitaxy.

Note, that the local curvature of the surface could impose a local concentration of the electric field during ECE and thus cause a non-uniform pattern of pores. Due to the fact that the star arms are higher than the surrounding area, the lines along the arms on the surface could be preferentially etched, but this is not the case, as confirmed in Figure 2. Moreover, the cross sections of the studied layers after ECE shown in Figure 3 confirm that the non-uniform pore pattern is related to the non-uniform electrical properties of GaN:Si layer in the layer volume at the nanoscale. If the geometrical factors were to play a role in preferential etching of the surroundings of the hillock arms, the cross section would be uniformly etched and this is not the case.

### Analysis of the Surface Morphology in Highly Si-Doped GaN

**Figure 4:**
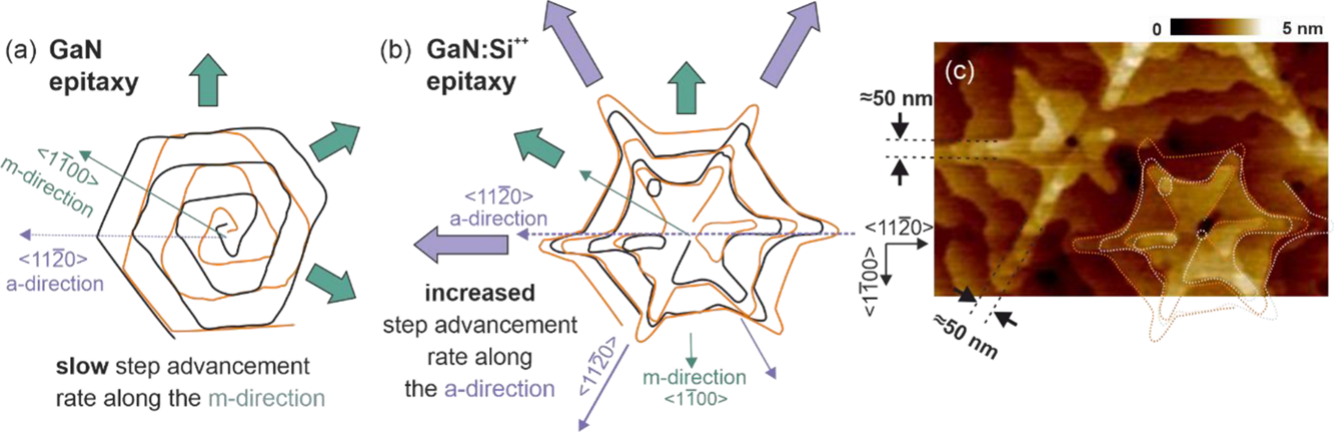
(a) Schematic step structure around hillock in GaN layer. The hillock is grown around a screw-type dislocation with a Burgers vector ***b*** = [0001]. Orange and black lines denote the step edges originating from a lattice shift of one atomic-plane distance. (b) Schematic picture of a step structure around the dislocation in GaN:Si^++^ during epitaxy by PAMBE under metal-rich conditions and (c) corresponding AFM image of highly doped GaN:Si *n* = 1 × 10^20^ cm^–3^. High Si doping enhances the growth along the *a*-direction that changes the hillock shape to be star-like.

Hexagonal shape hillocks were observed in undoped GaN layers grown by MBE^22,36^ and MOCVD^21,37^ using scanning tunneling microscopy (STM) or AFM. An example of a step-structure around a hillock is schematically depicted in Figure 4a, based on the work of Zheng et al.^36^ In this scheme, the hillock growth is related to the screw c-type dislocation of a Burgers vector ***b*** = [0001]. In the wurtzite structure, the consecutive crystal planes along the *c*-axis are rotated by 60° with respect to each other and spaced by 1/2 of the “*c*” vector. The slowest lateral step advancement rate defines the shape of the step-edges. In Figure 4a, the single atomic step edges are denoted by orange and black lines to present a double step structure that repeats every 120°. As a consequence of a different advancement rate of the step edges every 60°, the orange step is “faster than black” when it grows in the upward direction on the schematics and “slower than black” when it grows 60° left or right. The non-equivalent atoms arrangement on the step edges in GaN and its consequences for the surface morphology and incorporation of atoms have been discussed in more detail by Turski et al.^38^ and other authors.^22,37,39^

High Si doping strongly impacts the surface morphology of GaN:Si^++^ layers grown by MBE under metal-rich conditions. The shape of atomic step edges around the screw-type dislocation is significantly altered as schematically shown in Figure 4b. The drawing is prepared based on the AFM image of GaN:Si layer doped to the level of 1 × 10^20^ cm^–3^, presented in Figure 4c, that is a good representative for the most characteristic features of the atomic step edges shape around the hillocks in highly doped GaN:Si^++^ layers analyzed in this work. Both schemes in Figure 4a,b are prepared in the same orientation and for the hillock associated with the same dislocation Burgers vector length and direction. In the GaN:Si^++^ layer, the double step structure, the same as on a typical hillock, is clearly visible. The step-advancement rate along the *a*-direction ⟨112̅0⟩ is increased that makes the hillock change its shape from a hexagon to a star. A possible reason for this enhanced growth rate along the *a*-direction ⟨112̅0⟩ could be related to the enhanced diffusion. Higher growth rate along the *a*-direction should result in lower average Si incorporation,^3^ and it will be discussed in the following part of the paper.

### Evaluation of the Si Distribution by EDX

**Figure 5:**
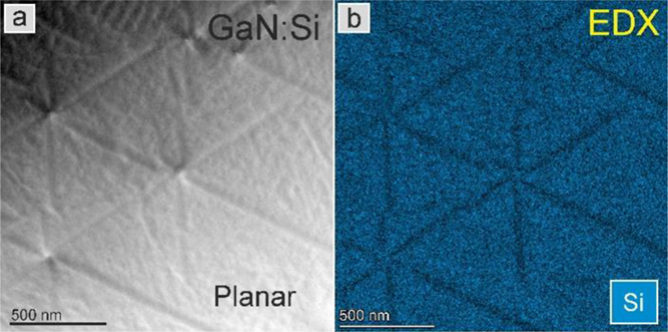
(a) HAADF image of the GaN:Si layer *n* = 1 × 10^20^ cm^–3^ and (b) corresponding EDX map of Si.

Plan view images of the GaN:Si layer *n* = 1 × 10^20^ cm^–3^ were studied in bright field (BF) and STEM mode using Z contrast with HAADF. These studies were combined with EDX mapping of Si (blue) and other elements such as N, Ga, Pt, and O. In the BF image shown in Figure 5a, the contrast form strain in the hillock arms is noted. Clearly, there is a correlation between the HAADF image and the EDX signal mapping the presence of Si, presented in Figure 5b. Quantification of the Si content in the arm was performed according to the approach verified previously.^33^ Average Si doping in the surrounding is taken as *n* = 1 × 10^20^ cm^–3^. The Si signal intensity measured within the star-arm area was compared with the reference Si signal intensity in the star surrounding. The line scans were extracted from the EDX maps collected on plan-view specimen shown in Figure 5, and the Si content in the nanostar arms was estimated to be 9.0 × 10^19^ cm^–3^. It corresponds to the difference of 10% in Si content between the star arm and the surrounding. The error bar in estimating the Si content is 0.2 × 10^19^ cm^–3^.

**Figure 6:**
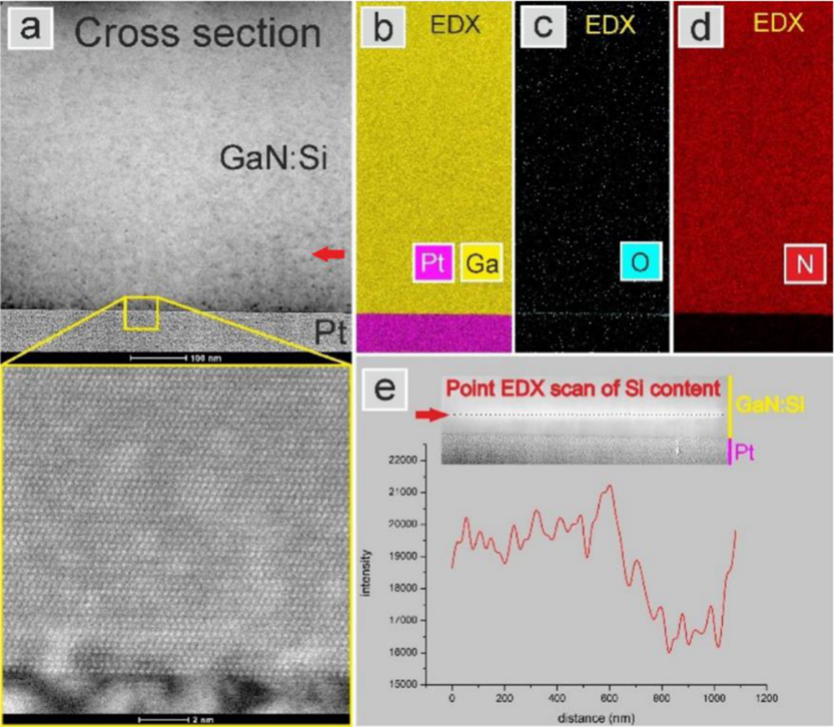
(a) HAADF images of GaN:Si layer *n* = 1 × 10^20^ cm^–3^ studied in cross section. The yellow box shows a magnification of the sample surface and its interface with Pt deposited on top. (b–d) EDX maps of the sample surface area showing the distributions of Ga, Pt, O, and N. (e) Line scan showing Si counts along the 1-μm-long distance with a visible drop in intensity that is attributed to the area of the star arm. The inset shows an array of the points where the EDX measurements were taken. The red arrow denotes the position of the line scan.

Similar quantification of Si signal intensity was done on the specimen prepared in cross section by FIB. The results of this measurement are presented in Figure 6. Due to the fact that there was no strain-related contrast within GaN:Si layer viewed in this projection, 1-μm-long line scan was performed at an arbitrary place as depicted in Figure 6e. The local intensity drop in the Si EDX signal corresponds to a decrease in the Si content at the ≈120 nm long distance, and the calculated Si content is 9.3 × 10^19^ cm^–3^. Despite that it was not possible to verify whether the cross section is done exactly along the star arm or along the arm that is inclined by 60° to the projection direction, very similar results are obtained in both approaches—plan view and cross section. Both measurements confirm that the increase in growth rate along the hillock arms imposes locally lower Si content.

### SSRM on Highly Doped GaN:Si

**Figure 7:**
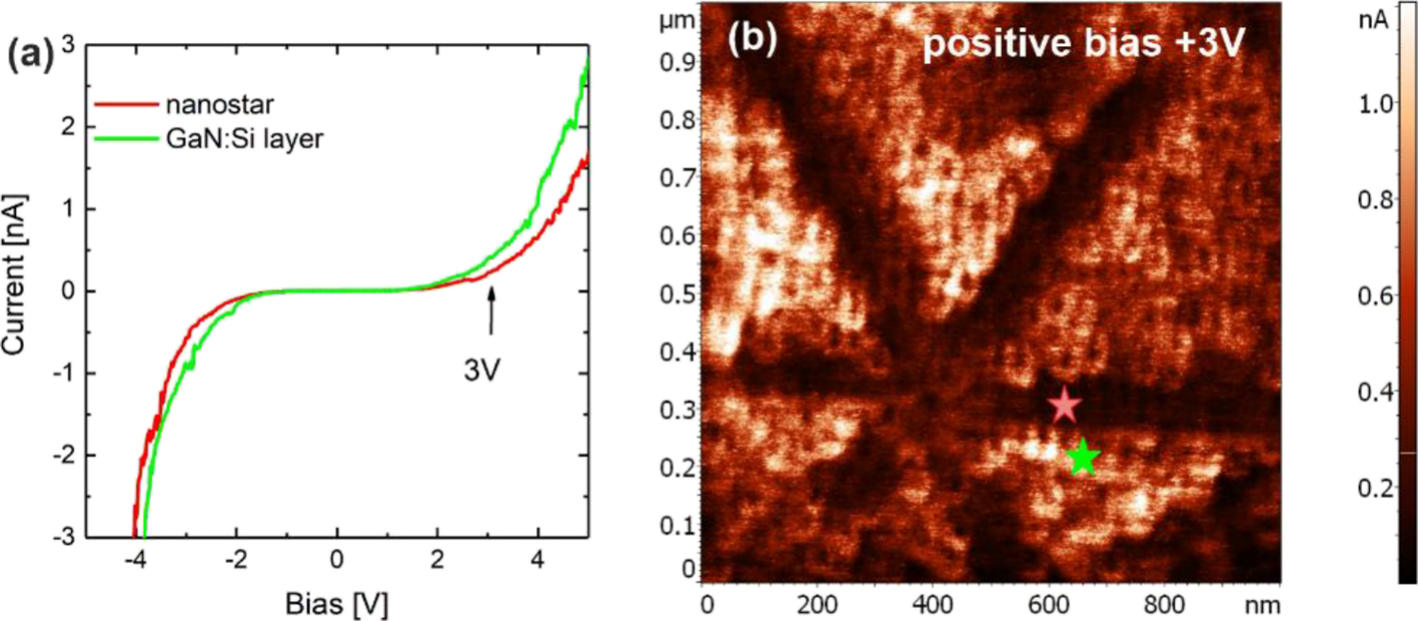
(a) Current–voltage (I–V) characteristics collected in the nanostar and in the surrounding GaN:Si layer. Measurement spots are schematically marked with red and green stars on the SSRM map in (b). (b) SSRM map collected for GaN:Si layer *n* = 1 × 10^20^ cm^–3^ applying a positive +3 V bias to the sample.

The local electrical properties of the highly doped GaN:Si layer have been studied by SSRM to check the local sample conductivity in relation to the surface morphology. Figure 7a presents the current–voltage (I–V) characteristics collected in the nanostar and in the surrounding GaN:Si layer measured in the range of −4 to +6 V. The I–V measurement points are schematically marked as stars in Figure 7b. A slightly lower current in the hillock arms is measured both when a positive or negative bias is applied to the sample. Figure 7b presents the current map collected on the GaN:Si layer doped to the level of 1 × 10^20^ cm^–3^ when a positive bias of +3 V is applied to the sample. Within a nanostar, a very low current ≈0.1 nA is measured. The higher current, 0.4 nA, is measured in the area between the arms.

The SSRM results confirm a noticeable non-uniform sample conductivity across the surface that correlates well with the topography and ECE results. Importantly, nanostars are not isolating. Both the nanostars and the surrounding material are conductive, but consistently a lower current, compared in absolute values, is measured in the nanostar.

## Discussion

Star-like hillocks are found in highly Si-doped MBE layers grown under metal-rich conditions. When the Si doping level is as high as 5 × 10^19^ and 1 × 10^20^ cm^–3^, the step advancement rate along the *a*-direction ⟨112̅0⟩ is enhanced, causing formation of nanostars, which are 50-nm-wide platelets of a material with lower conductivity, arranged in six-fold symmetry around the *c*-axis. Nanostars are associated with the growth on the screw-type-component threading dislocations. ECE of these highly doped GaN:Si layers reveals nanoscale inhomogeneity in electrical properties. The inhomogeneity is observed for the GaN:Si layers etched from the top and also laterally. Surprisingly, the nanostars were not etched electrochemically. The Si content in the star arms was estimated by high-resolution EDX studies to be only slightly lower, ≈10%, than in the surrounding layer. SSRM carried out on the highly doped GaN:Si layer confirmed that lower current is measured in the nanostar arms as compared to the surrounding material. It seems, however, that only the decrease of doping by 10% would not explain the results obtained by ECE, i.e., there was no visible etching of the nanostars. Because the ECE is sensitive to the doping level, one should expect slightly lower porosity in the star arm than in the rest of the material. Apparently, there is an additional effect that decreases the conductivity of the star arm. Therefore, we consider locally increased point defect density to be the compensation mechanism occurring at the nanoscale. An indication supporting the hypothesis that the hillock edges are prone to incorporate higher density of point defects is provided by cathodoluminescence (CL) studies on MOVPE^23^ and halide vapor phase epitaxy (HVPE)-grown GaN.^34^ Significantly lower CL intensity is seen only in the hillock edges that were seen as dark lines. Also Lee et al.^40^ have seen dark lines between the hillock facets in micro photoluminescence (PL) maps of GaN band-edge emission. The abovementioned studies discuss unintentionally doped GaN, so the probable point defects responsible for increased non-radiative recombination in the hillock arms are native vacancies or residual unintentional dopants such as oxygen, incorporating preferentially on the hillock edges due to the specific step-edge kinetics along the directions where the double steps interlace and change their arrangement. Note that gallium vacancies, *V*_Ga_, act as triple acceptors, effectively compensating Si donors. Also note that the presence of oxygen donor in GaN has been shown to promote the formation of Ga vacancies^41^ but the background oxygen doping level in MBE grown GaN is on the order of 10^16^ cm^–3^, so the excess formation of *V*_Ga_ does not seem to be favored.

When it comes to highly Si-doped layers, an additional compensation mechanism could take place. Theoretical works as well as experimental studies of AlGaN:Si grown by MOCVD suggest that the complexes of Ga vacancies with Si donors, Si_Ga_, could be the probable source of the significant drop in n-type doping due to the efficient compensation.^42−44^ Baker et al.^44^ suggest that for high enough Fermi level in the material, the formation of multiple-Si complexes with *V*_Ga_ becomes more favorable than the Si incorporating as substitutional atom onsite. Then, most of the donor switches from incorporating as onsite directly to incorporating as complexes. The authors explain that the three donor complexes, *V*_Ga_-3Si_Ga_, themselves are neutral and consume dopants rather than directly compensating them, with the majority of compensation arising instead from the two donor complexes, which act as acceptors. A recent study by Prozheev et al. also points out to this mechanism in GaN:Si grown by HVPE. Authors use positron annihilation and X-ray absorption near edge spectroscopy (XANES) to study Ga vacancies concentration in reference to the compensation in the material. They discuss the atomic-scale ordering of Si as a plausible reason for the reduced free carrier concentration.^45^ The effect of Si ordering while incorporating along the nanostar arms could possibly also take place in the highly doped GaN:Si layers grown by MBE. Therefore, although the concentration of Si is by 10% lower in the nanostars, the specific atom arrangement, i.e., ordering, in the lattice is a plausible reason for the decreased conductivity therein.

Note that the change in surface morphology of GaN:Si layers leading to the formation of nanostars is clearly noted only when the Si doping level is equal or higher than 6 × 10^19^ cm^–3^. There have been only a few reports of GaN:Si layers doped to such high levels grown by MBE that discuss surface morphology and structural properties. Lingaparthi et al.^46^ reported surface roughening for the GaN:Si^++^ layer *n* = 2 × 10^20^ cm^–3^ grown under slightly metal-rich conditions on Si(111) substrate as compared to the GaN:Si layers of lower doping analyzed in their study. High dislocation density in the substrate hindered the enhanced growth of hillock arms, but still the six-fold symmetry of the topography features can be noticed in AFM image although it is not commented by the authors. Zambrano-Serrano et al.^47^ also used Si(111) substrates with very high screw dislocation density of ≈1.6 × 10^10^ cm^–2^ and grew much thicker layers, 1.4 μm. They report mosaic structure for the GaN:Si^++^ layer of the highest doping *n* = 1.3 × 10^20^ cm^–3^. Mosaic structure was said to be produced by columnar grains with a random distribution of tilt angles. The surface morphology of this heavily doped layer is not explicitly shown, but other layers presented in this work seem to have much higher roughness than the ones studied in our work on GaN/sapphire templates. According to our best knowledge, the evolution of surface morphology from flat to nanostars with increasing Si doping has not been reported by other epitaxial techniques. It would be interesting to confirm this in experimental studies on MOCVD and HVPE grown GaN:Si as a function of their surface morphology and doping level. Interestingly, GaN layers grown by iodine vapor phase epitaxy^48^ exhibited star-shaped features, but their electrical properties were not discussed. Hillocks with flattened edges were also seen in undoped GaN layers grown by HVPE,^49^ but the length scale was much larger and again, authors did not consider inhomogeneity in electrical properties neither the mechanism responsible for such shape of the hillock edges.

Understanding the relation between the surface morphology during epitaxy and the resulting layer electrical properties is fundamental for the controlled device fabrication. For many applications, the inhomogeneity in Si incorporation of the order of 10% would be of negligible effect; however, the decrease in n-type doping by an additional compensation mechanism would be important. For instance, the inhomogeneity in electrical properties of GaN:Si layers grown on high dislocation density substrates hinders uniform porosification of these layers. This is a key to obtain high reflectivity of the porous DBR structures, such as the one shown in Figure 3d. Another important consequence of the presented results is the fact that the increased step-advancement rate along the *a*-direction ⟨112̅0⟩ that imposes the formation of nanostars in GaN:Si^++^ results in slightly increased surface roughness that is not welcome on the one hand. On the other hand, the specific morphology of highly doped GaN:Si layers could possibly offer also unexpected benefits such as could pave the way to the fabrication of InGaN-based emitters since the differences in local growth rate are expected to affect local indium incorporation.^20,50^

## Conclusions

In this work, we present the evidence that highly doped GaN:Si layers grown by MBE under metal-rich conditions on GaN/sapphire templates have non-uniform electrical properties at the nanoscale that are related to the specific surface morphology imposing inhomogeneous Si incorporation and local compensation. The growth rate in the *a*-direction ⟨112̅0⟩ is enhanced due to the high Si concentration at the surface during epitaxy. As a consequence, surface morphology changes and star-shaped hillocks become visible. Hillock density corresponds to the density of dislocations with a Burgers vector with a screw component. These star-shaped hillocks contain the nanostars, i.e., 50-nm wide platelets arranged in six-fold symmetry around the [0001] axis of a lower conductivity than the surrounding layer. The length of the nanostar arm is defined by local surface misorientation and is constrained by the neighboring hillocks. In the studied layers, it extends longer than 1 μm locally. The enhanced growth rate along the nanostars arms causes a locally lower Si concentration, which is estimated using EDX on a TEM specimen to be approximately 10% lower than in the surrounding layer. The electrical properties of the GaN:Si layer at the nanoscale are additionally studied using ECE and SSRM. Nanostars remain unetched by ECE in contrast to the GaN:Si layer, which becomes porous according to the applied bias. However, locally lower Si content within the nanostar cannot solely be responsible for the decrease in conductivity of the nanostars. SSRM confirmed lower conductivity of the nanostar arms. The compensation mechanisms that locally decrease the n-type conductivity in the nanostars are discussed: increased point defects incorporation, the formation of multiple Si complexes with vacancies, or the atomic-scale ordering of Si. Our study broadens the understanding of the relationship between the surface morphology observed in highly Si-doped GaN and the resulting electrical properties at the nanoscale.
